# Impact of nutritional supplementation on immune response, body mass index and bioelectrical impedance in HIV-positive patients starting antiretroviral therapy

**DOI:** 10.1186/1475-2891-12-111

**Published:** 2013-08-06

**Authors:** Denise Evans, Lynne McNamara, Mhairi Maskew, Katerina Selibas, Desiree van Amsterdam, Nicola Baines, Tracey Webster, Ian Sanne

**Affiliations:** 1 Health Economics and Epidemiology Research Office, Department of Internal Medicine, School of Clinical Medicine, University of the Witwatersrand, Johannesburg, South Africa; 2 Clinical HIV Research Unit, Department of Internal Medicine, Faculty of Health Sciences, University of the Witwatersrand, Johannesburg, South Africa; 3 Right to Care, Johannesburg, South Africa

**Keywords:** Nutritional supplement, Antiretroviral therapy, Human immunodeficiency virus, Treatment outcomes

## Abstract

**Background:**

Challenges to HIV care in resource limited settings (RLS) include malnutrition. Limited evidence supports the benefit of nutritional supplementation when starting antiretroviral therapy (ART) in RLS.

**Methods:**

Randomized controlled pilot study. HIV-positive ART-naive adults with self-reported weight loss were randomized to receive ART plus FutureLife porridge® nutritional supplement (NS) (388 kcal/day) or ART alone (Controls) for 6 months. Patients returned for monthly assessments and blood was drawn at enrolment and 6 months on ART. Differences in body composition, biochemical and laboratory parameters were estimated at 6 months on treatment.

**Results:**

Of the 36 randomized patients, 26 completed the 6 month follow-up (11 NS vs 15 Controls). At enrolment, groups were similar in terms of age, gender, body mass index (BMI) and bioelectrical impedance. NS patients had a lower median CD4 count (60 cells/mm^3^ [IQR 12–105 vs 107 cells/mm^3^ [IQR 63–165]; p = 0.149) and hemoglobin (10.3 g/dL [IQR 9.0-11.3] vs 13.1 g/dL [IQR 11.1-14.7]; p = 0.001).

At 6 months, NS patients increased their median CD4 count by 151 cells/mm^3^ [IQR 120–174) vs 77 cells/mm^3^ [IQR 33–145] in the Controls. NS patients had higher mean percentage change in body weight (12.7% vs 4.9%; p = 0.047), BMI (7.8% vs 5.5%; p = 0.007), absolute CD4 count (83.0% vs 46.4%, p = 0.002) and hemoglobin (9.5% vs 1.0%; p = 0.026). Patients in the NS arm had a higher mean percentage fat-free mass (16.7% vs −3.5%, p = 0.036), total body water (13.0% vs −1.9%, p = 0.026), intracellular water (16.1% vs −4.1%, p = 0.010) and basal metabolic rate (5.3% vs −0.2%, p = 0.014) compared to Controls. Patients in the NS arm also showed an improvement in physical activity at 6 months post-ART initiation compared to Controls (p = 0.037).

**Conclusion:**

Preliminary results are encouraging and suggest that NS taken concurrently with ART can promote weight gain, improve immune response and improve physical activity in HIV-positive patients that present at ART initiation with weight loss.

## Introduction

Malnutrition is a significant factor affecting human immunodeficiency virus (HIV) care and treatment in resource limited settings and contributes to other infections, including tuberculosis [1,2]. Individuals at all stages of HIV disease are at risk of nutritional deficiency and nutritional status is a strong predictor of disease progression, survival and functional status during the course of the disease [3]. The interplay between HIV/AIDS and malnutrition increases the burden of each condition alone. The HIV/AIDS pandemic in Africa has created a new form of vulnerability for households with regards to food security and nutrition. Poverty and food insecurity further threaten access to a nutrition-rich diet, hindering the chance of good health outcomes [4].

Adequate diet is believed to be important for adherence to antiretroviral therapy (ART) as inadequate nutrient intake is known to favour opportunistic infections and contributes to wasting. Individuals with HIV/AIDS require greater protein and micronutrient intake to support a weakened immune system [4] however they are also more vulnerable to malnutrition since they may have impaired nutrient absorption (due to diarrhoea/intestinal tract damage), reduced food intake (due to symptoms such as vomiting or pain on swallowing), food insecurity and medication side effects such as loss of appetite, depression or abdominal pain [5]. HIV and malnutrition may compound or cause severe immuno-deficiency which ultimately increases susceptibility to opportunistic infections [3]. Opportunistic infections can affect food intake, absorption and metabolism and so cause weight loss. Hence a cycle of infection, malnutrition and immuno-deficiency has been described [6].

Lack of essential micronutrients may not only contribute to the depletion and dysfunction of CD4 cells but malnourished patients may have a suboptimal response to treatment when (ART) is initiated [7]. Studies in industrialized countries before the advent of ART have shown a correlation between poor nutritional status at diagnosis and clinical progression of disease, but there is little data available from sub-Saharan Africa even though the two global epidemics of HIV and food scarcity affect the region disproportionally [8-10]. HIV-positive patients in southern Africa often present late for care with advanced immunosuppression and may already be affected by malnutrition (defined by the World Health Organization as a body mass index; BMI < 18.5 kg/m^2^) [11].

The benefits of ART on HIV-related survival are well-described and studies have shown that ART improves body mass index – a proxy for nutritional status [12-14]. Once ART has been established and malnutrition treated, the nutritional quality of the diet is important because of the long term metabolic effects of ART (dyslipidemia, insulin resistance, obesity) [15]. Weight loss and wasting have been predominant features of HIV disease progression and have long been established as strong predictors of mortality and morbidity in patients infected with HIV [16,17]. Routine assessment of HIV positive patients presenting for treatment has demonstrated repeated findings of decreased anthropometry and biochemical markers [3,18]. Furthermore, nutritional deficiencies of Vitamin B12, zinc and selenium in malnourished HIV-positive patients has been associated with decreased immunity and a higher risk of disease progression [19].

A variety of assessment tools can be used to determine nutritional status; these include anthropometric methods (i.e. skinfold measurement and mid-upper-arm circumference), BMI and bioelectrical impedance analysis (BIA). BIA allows researchers to determine the specific component of weight loss (fat-free mass [FFM], body cell mass [BCM] or fat mass [FM]) that is biologically relevant to the development of adverse outcomes. BCM refers to the cellular and metabolically active components of the body, including muscles, solid organs and intracellular water while FFM includes all the components of BCM plus additional components that are not metabolically active (extracellular water and tissue support) [20]. BIA is a rapid, affordable, non-invasive method for measuring changes in body composition and by doing so can predict poor prognosis in HIV-infected patients [20]. For example a low phase angle α (the relationship between two vector components of resistance and reactance) from bioelectrical impedance analysis has been shown to be an independent prognostic marker of clinical progression and survival in HIV-infected patients on ART [21,22]. Lower phase angles appear to be consistent with either cell death or a breakdown of the cell membrane whereas higher phase angles appear to be consistent with large quantities of intact cell membranes and body cell mass. A study amongst 539 HIV-positive adults with pulmonary tuberculosis from Kampala Uganda showed lower BMI, lower ICW:ECW, lower phase angle α and loss of body cell mass (BCM) and fat mass were associated with more advanced HIV infection (CD4^+^ lymphocyte count < 200 cells/mm^3^) [20]. Depletion of BCM has been associated with an increased risk of death among adults with HIV infection. BIA is a good tool for detecting body cell mass loss in HIV wasting and compares favorably with gold standard methods (total body potassium and dual energy x-ray absorptiometry) however its use has been limited by the lack of standardized methods and quality control procedures. Furthermore certain conditions must exist for measurements, for example, subjects should not have exercised or consumed alcohol within 8–12 hours of the test, subjects should not be moist from sweat or lotion and should not have a fever or be in shock.

Nutritional supplementation has been shown to boost overall health and energy, provide immune system support, increase efficacy of other medical treatment, improve general quality of life and support the healing process [15,23]. Nutritional interventions may help to strengthen the immune system and reduce the severity and impact of opportunistic infections [15]. Reducing weight loss could potentially have multiple benefits in terms of improving responses to ART or reducing mortality and morbidity. Despite this, evidence to support the benefit of nutritional supplementation on patients starting ART in settings with limited resources is scarce. Few studies have assessed the impact of food supplements and results from these studies suggest that nutritional supplementation is acceptable and feasible to improve food intake in malnourished HIV-infected patients [9,24].

We hypothesized that supplementation with additional calories and protein would improve nutritional status and possibly have an effect on body weight and immune status of HIV-positive patients starting ART. We investigate the effect of a nutritional supplement taken concurrently with ART for 6 months on body mass index and body composition as well as on laboratory and biochemical markers.

## Methods

### Study site and population

We conducted a randomized controlled pilot study at the Themba Lethu HIV Clinic at the Helen Joseph Hospital, a public-sector hospital in Johannesburg, South Africa between May 2010 and November 2011. This cohort has been described previously [25]. The pilot study was conducted to ensure there were no nutritional supplement-related adverse effects such as nausea, diarrhea or vomiting.

Forty-five antiretroviral naïve patients were recruited on the day they initiated standard first-line antiretroviral therapy (ART) according to the South African National Department of Health ART treatment guidelines [26]. Patients over 18 years with self-reported unintentional weight loss (defined as a drop in 2 dress sizes or loss of 5 – 10% of normal body weight in the last 3 months) were screened, consented and enrolled in the study. Pregnant women were excluded as were patients with an allergy to soya or iodine and those participating in any other supplemental feeding program.

Following patient screening and informed consent, study subjects were randomized 1:1 in permuted blocks of five to receive either ART plus FutureLife porridge® (FutureLife, P.O. Box 1035, New Germany, Durban 3620, South Africa) (NS) or ART alone (Control) for 6 months. Participants in the intervention arm were provided with, and instructed to consume, a high-protein high-energy meal (100 g equivalent to 388 cal/day), divided into several servings, concurrently with ART for 6 months (Table 1). FutureLife porridge® has been used in decentralized HIV programs in South Africa [24] and previous studies have also requested participants to consume 100 g/day or 400 cal/day of a nutritional intervention [27,28]. Compliance with the supplement was checked by questioning patients during their monthly visits.

**Table 1:** Components of the nutritional supplement

**Nutritional information**				
Nutrient	Size	Per 100 g	Per 50 g	% NRV per 50 g**
Energy	kJ	1673	837	*
Protein	g	18	9	16
Glycemic Carbohydrates	g	59	30	*
Of which total sugar	g	15	7.5	*
Total Fat of which	g	9.8	4.9	*
Saturated Fat	g	2.9	1.5	*
Monosaturated Fat	g	3.5	1.8	*
Polyunsaturated Fat	g	3.4	1.7	*
Trans fatty Acids	g	0.0	0.0	*
**Omega-3 Fatty Acids as:**				
Alpha-linolenic Acid (ALA)	mg	500	250	*
Omega 6 Fatty Acid	mg	3500	1750	*
Cholesterol	mg	0.0	0.0	*
Dietary Fibre	g	6.1	3	*
Total Sodium	mg	284	142	*
**Vitamins**				
Vitamin A (retinol)	μg	900	450	50
Vitamin B Complex	mg	1.2	0.6	50
B1 (Thiamine)	mg	1.2	0.6	50
B2 (Riboflavin)	mg	1.3	0.7	50
B3 (Nicotinic Acid)	mg	16	8	50
B5 (Pantothenic Acid)	mg	5	2.5	50
B6 (Pyridoxine)	mg	1.7	0.9	50
B9 (Folic Acid)	μg	400	200	50
B12 (Cobalamin)	μg	2.4	1.2	50
Vitamin C (Ascorbic Acid)	mg	100	50	50
Vitamin D (Cholecalciferol)	μg	15	7.5	50
Vitamin E (Tocopherol)	mg	15	7.5	50
Vitamin H (Biotin)	μg	30	15	50
Vitamin K	μg	120	60	50
**Minerals**				
Calcium	mg	337	167	13
Copper	mg	0.4	0.2	25
Iodine	μg	150	75	50
Iron	mg	18	9	50
Magnesium	mg	56	28	7
Manganese	mg	0.8	0.4	18
Molybdenum	μg	15	7.5	17
Phosphorus	mg	247	123	10
Selenium	μg	55	28	50
Zinc	mg	11	5.5	50
**Other**				
Inulin	mg	500	250	*
Lecithin	mg	500	250	*
Potassium	mg	455	228	*
MODUCARE®	μg	50000	25000	*
**Amino Acids**				
Alanine	mg	1110	555	*
Arginine	mg	1190	595	*
Aspartic Acid	mg	1480	740	*
Cysteine	mg	190	95	*
Glutamic Acid	mg	2830	1415	*
Glutamine	mg	1649	825	*
Glycine	mg	660	330	*
Histidine	mg	550	275	*
Isoleucine	mg	760	380	*
Leucine	mg	1360	680	*
Lysine	mg	1110	555	*
Methionine	mg	390	195	*
Phenylalanine	mg	970	485	*
Proline	mg	1070	535	*
Serine	mg	810	405	*
Threonine	mg	760	380	*
Tryptophan	mg	306	153	*
Tyrosine	mg	1050	525	*
Valine	mg	850	426	*
Allergens: Soya & milk/dairy – Sodium Caseinate (milk protein)				

* Nutrient Reference Value (NRV) not determined.

**% NRV nutrient reference value for individuals 4 years & older.

Both study arms received HIV care and treatment according to the South African National Department of Health ART treatment guidelines [26]. Patients returned to the clinic monthly for 6 months for clinical assessments (height, weight, blood pressure, bioelectrical impedance) and blood samples were collected at entry and 6 months. Patients received prospective serial assessment of nutritional intake (using a food frequency questionnaire and 5 day food recall diary), gastrointestinal (GI) symptoms, physical activity and adherence. BIA was measured at each monthly visit for 6 months using a Quantum BIA analyzer (RJL Systems, Clinton Township, Michigan, USA). A pregnancy test was performed at enrolment and at each visit thereafter. Participants that became pregnant during the study continued on the study, however BIA could not be performed on these participants.

Additional data for the study such as death, loss to follow-up (LTFU; defined as missing a clinic appointment by ≥ 3 months after last scheduled visit date), opportunistic infections and WHO stage were retrieved from the site’s electronic patient management system, TherapyEdge-HIV™. Laboratory data including CD4 count, hemoglobin and mean cell volume (MCV) at 12 months after ART initiation was also obtained from TherapyEdge-HIV™. All patients signed informed consent to take part in the study which, together with the use of TherapyEdge-HIV™ data, was approved by the University of the Witwatersrand Human Research Ethics Committee (Medical protocol M060623/M110140 and M090802).

### Study Variables

Blood samples were taken at enrolment and again at 6 months on treatment and included CD4 cell count, HIV-RNA viral load, MCV, hemoglobin, red blood cell, white blood cell and platelet count. Biochemical measurements included alanine transaminase (ALT), serum albumin, pre-albumin, iron (Fe), ferritin, % transferrin saturation, folate, magnesium (Mg), selenium and total calcium. Urine samples were collected at each visit for urine urea, creatinine and protein to monitor kidney function.

Height and weight together with bioelectrical impedance was collected for each patient at each monthly visit for 6 months. BMI, body fat percentage (% fat), FFM, lean dry mass (LDM), total body water (TBW), intra- and extra-cellular water (ICW, ECW), phase angle α, basal metabolic rate (BMR; measure of resting energy expenditure) and daily energy expenditure (DEE) were estimated using the RJL Body Composition 2.1 software from the resistance, reactance and patient details that were recorded (i.e. gender, height, weight, age, daily activity level and body frame size).

Physical activity was assessed at enrolment, 3 months and again at 6 months using a physical activity recall questionnaire. This included 16 multiple choice questions to assess an individual’s level of activity and calculate a physical activity score. In addition a 5 day physical activity recall diary using activity codes [29] and pedometer log were used to calculate calorie expenditure and verify physical activity level (PAL) [30]. Patient tolerance of the supplement and adherence to both ART and the supplement, was assessed at each monthly follow-up visit using an interview based questionnaire. Patients were asked if they experienced any adverse effects from ART or the supplement such as nausea, headaches, fever, pain, stomach cramps, skin rashes, vomiting, diarrhea, bloating, flatulence or insomnia. Adherence to both ART and the supplement was assessed using patient recall/self-reporting for the previous month. Patients were asked if they took the nutritional supplement or their antiretroviral (ARV) drugs every day for the past month, on average the number of days missed, the reason for not taking them and the number of tablets or the amount of supplement remaining at the end of the month.

### Statistical analysis

Patient demographics and clinical characteristics at ART initiation (baseline) were summarized and compared using T-test or Kruskal-Wallis for continuous data and Chi-square test for proportions. Results were presented using proportions or medians with interquartile ranges. Changes in body weight, BMI, bioelectrical impedance and biochemical, clinical and laboratory markers from ART initiation until 6 months follow-up were compared, by treatment arm, using paired T-test or Wilcoxon rank sum test for continuous data. Analyses were also adjusted for baseline CD4 cell count, hemoglobin, viral load, sex and age using repeated-measures analysis of variance (rANOVA). Changes in BIA and physical activity parameters were also stratified by level of immunodeficiency at study entry (< 100 vs. ≥ 100 cells/mm^3^). In addition, we assessed patient outcomes and changes in CD4 count, hemoglobin and MCV from ART initiation until 12 months after ART initiation, by treatment arm using data from TherapyEdge-HIV™). Patients were followed until the end of the study period (completers) but patients that prematurely discontinued study treatment (non-completers) (i.e. loss to follow-up, transferred out, died, withdrew) were excluded from the final analysis. Data was analyzed using SAS 9.3 (SAS Institute, Inc., Cary, NC, USA). A p value < 0.05 was considered significant in all analyses.

## Results

In total, 45 patients were screened and 38 patients were randomized (19 nutritional supplement/NS arm and 19 Control arm). Of the 7 patients that were not randomized, 1 patient did not have a valid SA ID document, 1 patient was admitted to hospital before he could be randomized and 5 patients did not return to the clinic.

Twenty six patients completed the 6 month follow-up visit (11 NS arm and 15 Control arm). On the NS arm, 3 patients transferred out, 3 patients withdrew from the study and 2 patients died. On the Control arm, 1 patient was LTFU, 1 patient transferred out and 2 patients withdrew from the study. The main reason for withdrawing from the study was work commitments (i.e. security guard on night shift). Non-completers were no different from completers in terms of age, gender or baseline BMI, hemoglobin, CD4 count and viral load (all p > 0.05), though more non-completers were initiated on TDF/3TC/NVP or d4T/3TC/EFV (p = 0.031) (Table 2). Patients that died or were LTFU were on ART for less than 30 days (median 28 days IQR 27 – 29) while patients that transferred out were on ART for a median of 63 days (IQR 23 – 119) compared to a median of 135 days (IQR 84 – 140) for those that withdrew.

**Table 2:** Patient demographics and baseline clinical characteristics of patients randomized to receive either ART plus nutritional supplement (NS) or ART alone (Control) for 6 months

**Characteristics**		**Nutritional supplement**	**Control**	**p value**	Completers^*^	Non-completers^*^	**p value**
		**n = 19**	**n = 19**		**n = 26**	**n = 12**	
Gender – Male	n,%	6 (31.6%)	7 (36.8%)	0.732	8 (30.8%)	5 (41.7%)	0.510
Age (years)	Median (IQR)	37 (32 – 42)	34 (30 – 43)	0.612	37 (32 – 42)	34.5 (31.5 – 42.5)	0.505
Employed	n,%	14 (73.7%)	11 (57.9%)	0.456	15 (57.7%)	10 (83.3%)	0.122
Education							
< Grade 8	n,%	2 (10.5%)	1 (5.3%)	0.222	1 (3.8%)	1 (8.3%)	0.114
≥ Grade 8	n,%	17 (89.5%)	18 (94.7%)		24 (92.3%)	11 (91.7%)	
Self-reported weight loss (kg)	Median (IQR)	5 (3 – 5)	5 (2 – 9)	0.094	5 (3 – 8)	5 (4 – 7)	0.356
Body mass index (BMI; kg/m^2^)	Median (IQR)	20.4 (18.0 – 22.4)	19.3 (18.4 – 21.3)	0.197	20.2 (17.6 – 21.7)	19.7 (18.0 – 21.7)	0.842
< 18.5 kg/m^2^	n,%	6 (31.6%)	4 (21.1%)	0.461	6 (23.1%)	1 (8.3%)	0.234
Fat (kg)	Median (IQR)	10.6 (10.0 – 17.7)	11.1 (5.5 – 15.5)	0.560	11.1 (9.1 – 15.9)	10.5 (8.6 – 12.0)	0.791
Systolic blood pressure (mmHg)	Median (IQR)	104 (90 – 125)	111 (107 – 127)	0.079	111 (102 – 127)	107 (95 – 120)	0.354
Diastolic blood pressure (mmHg)	Median (IQR)	69 (46 – 76)	71 (66 – 84)	0.209	71 (66 – 87)	68 (56 – 75)	0.209
WHO stage III/IV	n,%	4/12 (33.3%)	7/16 (43.8%)	0.611	7/19 (36.8%)	4/9 (44.4%)	0.966
ART regimen							
TDF/3TC/EFV	n,%	14 (73.7%)	14 (73.7%)	1.000	22 (84.6%)	6 (50.0%)	0.031
TDF/3TC/NVP	n,%	1 (5.3%)	1 (5.3%)		-	2 (16.7%)	
d4T/3TC/EFV	n,%	4 (21.0%)	4 (21.0%)		4 (15.4%)	4 (33.3%)	
CD4 cells/mm^3^	Median (IQR)	60 (12 – 105)	107 (63 – 165)	0.149	86 (24 – 125)	99 (70 – 121)	0.405
≤ 50	n,%	8 (42.1%)	4 (21.0%)	0.027	10 (38.5%)	2 (16.7%)	0.152
51 – 100	n,%	5 (26.3%)	5 (26.4%)		6 (23.0%)	4 (33.3%)	
101 – 200	n,%	6 (31.6%)	6 (31.6%)		6 (23.0%)	6 (50.0%)	
> 201 – 350	n,%	0 (0.0%)	4 (21.0%)		4 (15.5%)	0 (0.0%)	
Hemoglobin (g/dL)	Median (IQR)	10.3 (9.0 – 11.3)	13.1 (11.1 – 14.7)	0.001	11.6 (10.3 – 13.6)	11.0 (9.4 – 12.6)	0.294
< 10 g/dL	n,%	9 (47.4%)	1 (5.3%)	0.009	5 (19.2%)	5 (41.7%)	0.144
Viral load (log10)	Median (IQR)	5.4 (4.7 – 5.8)	5.6 (5.4 – 6.0)	0.042	5.5 (4.7 – 5.8)	5.2 (4.9 – 5.6)	0.851
Mean cell volume (fL)	Median (IQR)	85.0 (78.4 – 98.9)	91.3 (84.0 – 94.8)	0.090	90.1 (81.5 – 94.6)	83.2 (78.3 – 88.0)	0.096
TB at initiation	n,%	2 (10.5%)	0 (0.0%)	0.157	1 (4.4%)	1 (8.3%)	0.629
Alcohol (current/history)	n,%	8 (42.1%)	4 (21.1%)	0.045	8 (30.8%)	4 (33.3%)	0.846
Smoking (current/history)	n,%	5/19 (26.3%)	1/18 (5.6%)	0.063	3/26 (11.5%)	2/11 (18.2%)	0.409

*Patient demographics and baseline clinical characteristics of those that completed (completers) and those that did not complete the 6 month follow-up (non-completers) were compared.

### Baseline demographics and clinical characteristics

Patients in the NS arm were similar to those in the control arm in terms of age, gender, education and employment (Table 2). Patients in the NS arm had a lower median CD4 count (60 cells/mm^3^ IQR 12 – 105 vs. 107 cells/mm^3^ IQR 63 – 165; p = 0.149), lower median hemoglobin (10.3 g/dL IQR 9.0 – 11.3 vs. 13.1 g/dL IQR 11.1 – 14.7; p = 0.001) and higher median body mass index (20.4 kg/m^2^ IQR 18.0 – 22.4 vs. 19.3 kg/m^2^ IQR 18.4 – 21.3; p = 0.197) compared to those in the Control arm. A greater proportion of patients in the NS arm had a CD4 ≤ 50 cells/mm^3^ (42% vs. 21%; p = 0.027) together with a current or prior history of alcohol use (p = 0.029). Patients in the NS arm had higher physical activity scores at treatment initiation than those in the Control arm (median physical activity score 17 IQR 14 – 23 vs. 14 IQR 12 – 17; p = 0.024). The score is the inverse of the physical activity so patients in the NS arm had a higher score but lower physical activity compared to the Control arm.

### Percentage change in clinical characteristics and biochemistry

At 6 months on ART, patients in the NS arm demonstrated a greater increase in weight (12.7% vs. 4.9%; p = 0.047), BMI (7.8% vs. 5.5%; p = 0.007), CD4 count (83.0% vs. 46.4%; p = 0.002) and hemoglobin (9.5% vs. 1.0%; p = 0.026) when compared to Controls (Table 3). 80% (8/10) of patients in the NS arm compared to 40% (6/15) in the Control arm showed an increase in CD4 of ≥ 100 cells/mm^3^ at 6 months (p = 0.048). Likewise, patients in the NS arm increased their CD4 count by 151 cells/mm^3^ vs. 77 cells/mm^3^ in the Control arm (p = 0.024).

**Table 3:** Median (IQR) for anthropometric indices, biochemical and laboratory markers at baseline, 6 months and the median (IQR) % change calculated between baseline and 6 months follow-up

	**Nutritional supplement**			**Control**			
	**Baseline**	**6 months**	**% change**^**#**^	**Baseline**	**6 months**	**% change**^**#**^	**p value**
	**N = 19**	**N = 11**		**N = 19**	**N = 15**		
**Clinical characteristics**							
Weight (kg)	53.4 (45.6 – 59.0)	59.6 (48.0 – 65.4)	12.7 (7.0 – 25.3)	52.8 (48.7 – 60.4)	54.0 (50.2 – 61.3)	4.9 (−1.6 – 11.2)	0.047
Body mass index (BMI; kg/m^2^)	20.4 (18.0 – 22.4)	22.1 (19.9 – 24.3)	7.8 (7.0 – 14.4)	19.3 (18.4 – 21.3)	20.0 (18.5 – 23.7)	5.5 (0.5 – 10.9)	0.007
Systolic blood pressure (mmHg)	104 (90 – 125)	111 (100 – 119)	6.7 (−10.1 – 35.6)	111 (107 – 127)	118 (99 – 134)	−0.8 (−7.5 – 10.0)	0.273
Diastolic blood pressure (mmHg)	69 (46 – 76)	70 (59 – 75)	2.9 (−22.2 – 45.0)	71 (66 – 84)	73 (63 – 88)	0.1 (−7.6 – 4.5)	0.685
CD4 (cells/mm^3^)	60 (12 – 105)^*^	167 (154 – 293)	83.0 (76.4 – 93.1)	107 (63 – 165)^*^	233 (152 – 359)	46.4 (20.7 – 60.4)	0.002
≤100 cells/mm^3^	34 (12 – 60)	157 (107 – 246)	88.9 (79.3 – 95.3)	63 (35 – 86)	189 (100 – 251)	56.9 (49.0 – 81.6)	0.039
>100 – 350 cells/mm^3^	117 (105 – 135)	259 (225 – 293)	56.6 (53.8 – 59.4)	148 (125 – 257)	324 (233 – 377)	21.1 (20.4 – 34.8)	0.018
Hemoglobin (g/dL)	10.3 (9.0 – 11.3)^*^	12.5 (11.1 – 13.6)	9.5 (1.1 – 40.4)	13.1 (11.1 – 14.7)^*^	13.6 (12.8 – 14.7)	1.0 (−1.5 – 7.6)	0.026
Mean cell volume (fL)	85.0 (78.4 – 89.9)	93.7 (81.7 – 96.4)	6.4 (4.7 – 7.2)	91.3 (84.0 – 94.8)	99.3 (91.8 – 103.4)	8.1 (3.7 – 9.0)	0.608
Red blood cells (x10^12^/L)	3.7 (3.1 – 3.9)^*^	4.1 (3.7 – 4.1)	7.0 (−4.8 – 34.3)	4.3 (3.9 – 4.9)^*^	4.1 (3.7 – 4.6)	−4.7 (−11.3 – 3.8)	0.043
White blood cells (x10^9^/L)	3.4 (2.7 – 5.1)	4.5 (3.2 – 4.7)	28.6 (21.6 – 52.4)	4.4 (3.3 – 5.5)	3.7 (3.4 – 5.0)	−2.8 (−34.2 – 29.3)	0.035
Platelets (x10^9^/L)	277 (210 – 326)	272 (235 – 322)	11.1 (−16.3 – 23.1)	231 (152 – 351)	256 (184 – 362)	5.6 (−14.9 – 21.1)	0.787
**Anthropometric indexes**							
Fat (kg)	10.6 (10.0 – 17.7)	12.8 (11.4 – 22.9)	23.3 (−6.3 – 43.9)	11.1 (5.5 – 15.5)	11.9 (9.5 – 20.6)	18.4 (10.2 – 80.5)	0.375
Fat Free Mass (FFM; kg)	38.0 (34.2 – 43.1)	42.5 (35.8 – 50.9)	16.7 (2.7 – 25.8)	41.7 (39.1 – 47.1)	39.8 (35.5 – 43.1)	−3.5 (−9.9 – 4.1)	0.036
Total Body Water (TBW; kg)	27.2 (23.5 – 30.4)	27.5 (24.1 – 36.4)	13.0 (2.6 – 26.1)	30.0 (25.8 – 36.3)	27.8 (24.2 – 31.3)	−1.9 (−15.6 – 5.9)	0.039
Intracellular Water (ICW; kg)	14.4 (12.5 – 17.3)	15.9 (13.2 – 21.3)	16.1 (4.3 – 28.5)	17.9 (14.7 – 19.2)	15.9 (13.1 – 18.8)	−4.1 (−15.3 – 1.6)	0.011
Extracellular Water (ECW; kg)	12.0 (10.6 – 14.1)	12.9 (11.5 – 15.9)	1.6 (3.8 – 23.7)	12.5 (10.9 – 16.6)	11.7 (10.8 – 15.1)	−1.3 (−16.6 – 2.6)	0.028
Phase angle	6.1 (5.1 – 6.9)	6.6 (6.2 – 7.3)	25.9 (4.8 – 39.4)	6.8 (6.3 – 7.3)	6.7 (5.9 – 7.8)	−1.5 (−10.8 – 25.8)	0.063
**Biochemistry**							
Albumin (g/L)	35 (33 – 37)	40 (39 – 42)	15.6 (5.4 – 20.0)	36 (32 – 42)	42 (39 – 42)	11.8 (−2.3 – 25.7)	0.700
Pre-albumin (g/L)	0.22 (0.16 – 0.24)	0.26 (0.23 – 0.28)	21.7 (16.7 – 38.9)	0.21 (0.14 – 0.25)	0.27 (0.25 – 0.34)	18.0 (4.5 – 54.2)	0.892
Alanine transaminase (ALT;IU/L)	29 (15 – 35)	33 (19 – 41)	−15.2 (−40.4 – 90.0)	26 (19 – 38)	35 (22 – 84)	36.4 (7.7 – 117.6)	0.173
Calcium (mmol/L)	2.2 (2.0 – 2.2)	2.2 (2.2 – 2.3)	3.6 (−0.5 – 9.3)	2.2 (2.0 – 2.3)	2.2 (2.1 – 2.3)	3.9 (−3.0 – 7.9)	0.766
Selenium (ug/L)	64 (52 – 79)	81 (69 – 116)	8.9 (−11.1 – 150.4)	71 (53 – 86)	95 (78 – 112)	38.1 (−6.7 – 86.8)	0.968
Magnesium (mmol/L)	0.76 (0.74 – 0.82)	0.77 (0.73 – 0.83)	2.7 (−3.9 – 9.2)	0.76 (0.70 – 0.84)	0.76 (0.73 – 0.82)	0.0 (−2.6 – 4.3)	0.643
Iron (umol/L)	8.2 (5.4 – 11.9)	11.3 (6.7 – 11.8)	1.3 (−42.2 – 79.7)	10.8 (6.9 – 16.1)	13.2 (12.1 – 18.9)	10.9 (−23.7 – 57.7)	0.558
Ferritin (ug/L)	329 (162 – 746)	26 (23 – 100)	−90.3 (−96.9 – -50.3)	141 (58 – 366)	55 (23 – 76)	−48.9 (−79.3 – -36.8)	0.045
Saturation transferrin (%)	16 (8 – 23)	13 (10 – 18)	−27.3 (−68.6 – 37.5)	17 (9 – 22)	20 (16 – 22)	0.0 (−22.7 – 38.5)	0.244
Folate (ug/L)	24 (23 – 35)	23 (7.0 – 35)	−0.4 (−67.8 – 34.2)	26 (23 – 38)	21 (10 – 24)	−43.6 (−71.6 – 6.0)	0.443
Urine urea (mmol/L)	267 (139 – 396)	271 (213 – 313)	6.9 (−67.9 – 205.3)	155 (97 – 244)	244 (185 – 322)	45.1 (−19.5 – 258.3)	0.484
Urine creatinine (mmol/L)	11 (8 – 14)	9 (8 – 12)	−20.0 (−46.3 – 13.1)	9 (5 – 13)	9 (7 – 10)	20.0 (−36.9 – 97.7)	0.334
Urine protein (g/mmol)	0.28 (0.18 – 0.63)	0.21 (0.17 – 0.39)	−43.3 (−86.2 – -18.2)	0.20 (0.11 – 0.47)	0.19 (0.10 – 0.24)	−41.7 (−61.5 – 81.8)	0.459
**Physical activity/Energy expenditure**							
Physical activity score	17 (14 – 23)^*^	12 (11 – 13)	−37 (−52 – -14)	14 (12 – 17)^*^	11 (10 – 18)	−10 (−23 – 10)	0.037
Basal Metabolic Rate (BMR)	1273 (1205 – 1345)	1312 (1226 – 1481)	5.3 (3.2 – 12.3)	1357 (1244 – 1402)	1365 (1218 – 1429)	−0.2 (−1.0 – 2.7)	0.014

*Significant difference between NS and Control group at baseline (p < 0.05); ^#^Median (IQR) of the differences (% change) between baseline and 6 months. The percentage change was compared between the two arms, nutritional supplement and Control arm.

Patients in the NS arm also showed an increase in red blood cells (7.0% vs. -4.5%; p = 0.043) and white blood cells (28.6% vs. -2.8%; p = 0.035) when compared to the Control arm. Serum ferritin was the only biochemical marker that showed a significant change between the two arms, patients in the NS arm had a decrease in serum ferritin compared to the Control arm (−90.3% vs. -48.9%; p = 0.045).

In analyses adjusted for baseline CD4 cell count, haemoglobin, viral load, sex and age, the supplement group showed significant increases in weight (p = 0.054), BMI (p = 0.028), CD4 (p = 0.002), hemoglobin (p = 0.053), white blood cells (p = 0.051) and red blood cells (p = 0.015).

### Change in bioelectrical impedance

Percentage change in bioelectrical impedance from ART initiation until 6 months follow-up was calculated and compared. Percentage change in FFM (16.7% vs. -3.5%; p = 0.036), TBW (13.0% vs. -1.9%; p = 0.039), ICW (16.1% vs. -4.1%; p = 0.011), ECW (1.6% vs. -1.3%; p = 0.028) and BMR (5.3% vs. -0.2%; p = 0.014) were all higher in the NS arm compared to the Control arm. These results were confirmed in a sub-analysis where only patients in the NS arm with a CD4 count ≥ 100 cells/mm^3^ showed a significant increase in FFM (p = 0.025), ICW (p = 0.016), ECW (p = 0.015) and BMR (p = 0.040) (Table 4).

**Table 4:** Changes in BIA and physical activity parameters at 6 months, by level of immunodeficiency at study entry (baseline)

**Percentage change at 6 months from baseline, by CD4 count**							
	**Nutritional supplement**			**Control**			
**Parameter**	**Baseline**	**6 months**	% change^*^	**Baseline**	**6 months**	**% change**	**p value**
**< 100 cells/mm**^**3**^	N = 13			N = 9			
Fat Free Mass (FFM; kg)	37.4 (34.2 – 43.2)	41.2 (35.6 – 51.7)	14.1 (1.6 – 19.2)	41.7 (39.1 – 47.1)	40.2 (35.7 – 42.9)	3.9 (−11.0 – 7.3)	0.811
Total Body Water (TBW; kg)	26.8 (23.5 – 31.4)	29.4 (24.9 – 36.8)	10.9 (2.5 – 21.1)	29.5 (26.5 – 36.3)	28.5 (25.2 – 30.9)	1.4 (−18.5 – 15.1)	0.806
Intracellular Water (ICW; kg)	14.3 (12.5 – 18.4)	16.6 (13.7 – 21.5)	13.6 (2.0 – 21.5)	16.8 (14.7 – 18.9)	15.8 (13.9 – 17.5)	1.6 (−18.0 – 15.7)	0.804
Extracellular Water (ECW; kg)	11.4 (10.6 – 14.1)	12.9 (11.5 – 15.4)	7.8 (3.0 – 19.9)	13.2 (11.3 – 17.2)	12.4 (11.6 – 14.2)	0.6 (−20.5 – 14.3)	0.805
Basal metabolic rate (BMR)	1264 (1205 – 1393)	1291 (1229 – 1520)	4.1 (2.4 – 9.2)	1348 (1285 – 1379)	1349 (1255 – 1403)	2.4 (−0.4 – 9.2)	0.179
Physical activity score	15.0 (14.0 – 20.0)	11.0 (10.5 – 12.5)	−38.7 (−51.1 – -22.5)	14.0 (13.0 – 16.0)	11.5 (10.5 – 16.0)	−8.9 (−25.9 – 9.8)	0.028
**≥ 100 cells/mm**^**3**^	N = 6			N = 10			
Fat Free Mass (FFM; kg)	42.0 (25.5 – 42.8)	42.5 (36.3 – 44.2)	17.4 (5.0 – 29.8)	42.3 (37.8 – 50.0)	39.8 (35.5 – 52.4)	−5.4 (−15.3 – -0.8)	0.025
Total Body Water (TBW; kg)	30.1 (19.1 – 30.4)	24.0 (12.0 – 32.7)	13.7 (7.0 – 20.4)	30.8 (25.0 – 36.5)	27.3 (24.2 – 38.6)	−8.2 (−18.7 – 0.4)	0.143
Intracellular Water (ICW; kg)	16.0 (9.6 – 16.7)	15.9 (13.2 – 16.8)	16.0 (4.8 – 27.3)	18.6 (15.0 – 21.3)	16.8 (12.6 – 22.9)	−7.9 (−18.3 – -2.2)	0.016
Extracellular Water (ECW; kg)	13.4 (9.5 – 14.4)	14.6 (10.8 – 15.9)	10.7 (9.4 – 12. 0)	12.0 (10.7 – 15.8)	11.6 (10.5 – 15.6)	−3.2 (−19.9 – 0.9)	0.015
Basal metabolic rate (BMR)	1292 (1092 – 1345)	1418 (1226 – 1441)	8.0 (5.1 – 10.9)	1379 (1205 – 1471)	1365 (1190 – 1512)	−0.8 (−1.6 – 1.2)	0.040
Physical activity score	19.5 (16.0 – 28.0)	13.0 (12.0 – 16.0)	−36.8 (−53.6 – 33.3)	13.5 (12.0 – 17.0)	11.0 (10.0 – 18.0)	−20.0 (−23.1 – 10.0)	0.630

*Median (IQR) of the differences (% change) between baseline and 6 months. The percentage change was compared between the two arms, nutritional supplement and Control arm.

### Physical activity, adherence and tolerability

When using the physical activity score, patients in the NS arm showed an improvement in physical activity at 6 months post-ART initiation compared to those in the Control arm (p = 0.037). In a sub-analysis, only patients with baseline immunodeficiency (CD4 < 100 cells/mm^3^) showed a significant improvement in physical activity (p = 0.028) (Table 4).

At 6 months, a greater proportion of patients in the NS arm reported improvements in their physical activity level (PAL 11/11 vs. 9/15; p = 0.057), weight (10/11 vs. 8/15; p = 0.095) and appetite (9/11 vs. 10/14; p = 0.424) compared to those in the Control arm. The nutritional supplement did not significantly contribute to adverse side effects in terms of depression (18% vs. 53%; p = 0.067), body pain or headaches (18% vs. 53%; p = 0.067), nausea (0% vs. 13%; p = 0.208), fever (18% vs. 27%; p = 0.612), skin rashes (27% vs. 33%; p = 0.740), stomach cramping (10% vs. 27%; p = 0.262), vomiting or diarrhoea (18% vs. 13%; p = 0.735), bloating or flatulence (10% vs. 13%; p = 0.735) or insomnia (0% vs. 20%; p = 0.115). During the course of the study, 2 patients were diagnosed with Herpes zoster (WHO stage II condition; 1 patient on each arm) while 1 patient on the NS arm was diagnosed with peripheral neuropathy on tenofovir with lamivudine and efavirenz.

Tolerance of, and adherence to, the NS was good during the study period. 82% (9/11) of patients in the NS arm reported taking the supplement every day for the previous month. Patients on both the NS arm (11/11; 100%) and the Control arm (14/15; 93%) reported good ART adherence (defined as taking 90% or more of their ARVs in the previous month; p = 0.383).

### Patient outcomes and changes at 12 months after ART initiation

Twenty patients were still alive and in care 12 months after ART initiation (9 NS arm and 11 Control arm). On the NS arm, 1 patient transferred out (196 days) and a second was LTFU (428 days). On the Control arm, 2 patients transferred out (median 393 days IQR 372 – 415) and 2 patients were LTFU (median 416 days IQR 378 – 454).

The difference in CD4 count increase between the two arms observed at 6 months did not continue after the completion of the study, where the supplement was stopped and both arms continued on ART alone. The % change between 6 and 12 months after ART initiation was 41.6% (IQR 36.9 – 97.0) for the NS arm and 31.5% (IQR −15.7 – 54.2) for the Control arm (p = 0.309). However the difference in BMI increase between the two arms was still evident 12 months after ART initiation, even after patients on the NS arm had stopped taking the supplement. The % increase at 12 months was 4.2% (IQR 3.9 – 12.0) for the NS arm compared to 0.2% (IQR −15.7 – 54.2) on the Control arm (p = 0.046).

## Discussion

Adequate nutritional status supports immunity and physical performance [15]. Weight loss, caused by low dietary intake, malabsorption and altered metabolism, is common in HIV infection. Addressing poor nutritional status may, therefore, improve clinical outcomes in HIV-infected individuals by reducing the incidence of HIV-associated complications and attenuating progression of HIV disease, thereby improving quality of life and ultimately reducing disease-related mortality [23].

We demonstrate that nutritional supplementation taken concurrently with ART for 6 months resulted in an increase in BMI, CD4 count, hemoglobin, red blood cell and white blood cell count and improvement in physical activity when compared to Controls. In addition, serum ferritin was the only biochemical marker that was significantly different between the two arms. Patients in the NS arm showed an increase in FFM, TBW, ICW, ECW and BMR when compared to Controls. Phase angle α was also higher in patients in the NS arm (26% vs. -1.5%; p = 0.063) which may suggest improved health in these patients which may result in improved treatment outcomes. By 12 months after ART initiation, patients in the NS arm continued to show a significant increase in BMI but not CD4 count when compared to the Control arm. The greatest gain in CD4 count and improvement in physical activity was observed in patients in the NS arm with a CD4 count < 100 cells/mm^3^ at study entry.

Baseline characteristics between the NS and the Control arm were similar although patients in the NS arm were slightly older with a lower CD4 count and hemoglobin. These patients also had a lower viral load, higher BMI and a greater improvement in physical activity level compared to those in the Control arm. These differences are likely due to chance and not the incorrect randomization of sicker or thinner patients to the NS arm.

We observed an improvement in various parameters in the intervention arm. Patients in the NS arm demonstrated an increase in body weight, BMI, CD4 count and hemoglobin. These results suggest that nutritional supplementation for 6 months led to an improved recovery of the immune system and an improvement in the body’s ability to fight infections. This also supports reports that adequate nutrition promotes and maintains optimal immune function [23]. Fawzi and co-workers (2004) reported an increase in CD4 (48 x 10^6^ cells/L IQR 10 - 85) in their study using a multivitamin supplementation [27,31,32] whereas Sattler and co-workers (2008) and Swaminathan and co-workers (2010) observed a slowing decline in immune function. A possible explanation for the difference observed may be that the improvement in CD4 count is related to regression-to–the-mean since the baseline counts were lower in the NS arm, albeit not statistically significant. Regardless, there are potential benefits of supplementation in immune status for patients with active weight loss and severe immunosuppression. Unlike the study by de Luis and colleagues (2001) we did not continue to observe a significant difference in percentage CD4 cell count between the 2 arms after the completion of the study [33].

Studies have reported that ART improves BMI while nutritional supplementation further increases BMI [15,27,34,35]. We continued to observe a difference in BMI between the 2 arms 6 months after the completion of the study or 12 months after ART initiation. Regaining weight, particularly muscle mass, requires ART, treatment of opportunistic infections, consumption of a balanced diet, physical activity and mitigation of side effects [15]. Studies have also shown an association between early weight gain when receiving ART and improved treatment outcomes [10]. Reports suggest that there may be an optimal pre-treatment BMI range for immune recovery on ART. Patients with lower BMI with a weight change of < 10% during follow-up show markedly reduced CD4 count recovery, suggesting that a failure to gain needed weight may be a marker of incomplete virologic suppression, an intercurrent illness or may preclude an optimal response to ART [7]. We did not find a significant increase in body fat between the two arms. Treatments that induce an increase in body fat, without affecting lean mass, do little to improve nutrition-related outcomes, such as functional status, quality of life and disease progression but may contribute to increased cardiovascular disease risk or hypertension [31].

The effect of a specific micronutrient will depend not only on the background intake of the micronutrient given, but also on the intake of other interacting micronutrients. To assume that all study participants are initially deficient with respect to one or more micronutrients would be incorrect [2]. Serum ferritin was the only parameter that showed a statistical decrease in the NS arm compared to the Control arm - studies have shown a high serum ferritin concentration in patients with anemia [36]. A significant increase in hemoglobin and red blood cells and a significant decrease in serum ferritin may suggest a decrease in the severity of anemia in the NS arm. Serum ferritin can be influenced by other factors other than iron status so another possibility is that supplementation reduces generalized inflammation. Serum ferritin, accompanied by normal or even decreased transferrin saturation, is elevated by inflammation and tissue damage. Dunn-Lewis and colleagues showed that a multi-nutrient supplement effectively reduced inflammatory status in both men and women [37]. It has been suggested that decreasing inflammation directly in the gut may result in an immunologic benefit, possibly by promoting growth of indigenous microflora or “good” bacteria [38].

Significant increases in FFM, TBW, ICW and ECW were observed in the supplement arm compared to the Control arm, findings similar to those reported by Swaminathan and colleagues (2010) and Schwenk and colleagues (1999) [27,39]. The number of patients on the NS arm with a low phase angle α (< 5.3) reduced from 6 at ART initiation to zero (0) at 6 months. This may result in better clinical outcomes since a low phase angle α has been correlated with the disease progression in HIV-infection and increased risk of morbidity or mortality [21,22].

As expected, and consistent with other reports, patients with a CD4 count < 100 cells/mm^3^ have lower median values for bioelectrical impedance at ART initiation [18]. Reports show that absolute gains in weight, BMI, CD4 cell count, FFM and BCM are higher among patients with severe immunodeficiency, indicating that demonstrable improvements are most likely to be seen among the most immunosuppressed [3,21]. However, we demonstrate that gains in FFM, ICW, ECW and BMR were greater in patients with CD4 cell counts ≥ 100cells/mm^3^. Swaminathan and colleagues (2010) found that gains were greater in patients with CD4 cell counts < 200cells/mm^3^. A possible explanation for the lack of effect seen in severely immunosuppressed patients (CD4 < 100 cells/mm^3^) may be that ART needs to be established first before malnutrition can be treated or before protein synthesis and immune-related enzyme systems can resume their functions [18]. As previously mentioned, regaining weight requires ART and treatment of opportunistic infections, consumption of a balanced diet, physical activity and mitigation of side effects [15]. Interestingly, severely immunosuppressed patients (CD4 < 100 cells/mm^3^) in the intervention arm showed the greatest improvement in physical activity.

This is one of a few studies investigating a nutritional supplement intervention that compares attrition across 2 groups of HIV-positive patients. Long term follow-up provided the opportunity to comment on patient outcomes 6 months after the completion of the study (12 months after ART initiation). Very few studies have assessed the impact of food supplements or reported on mortality, morbidity or disease progression [15,23]. Only 2 deaths were recorded, both on the nutritional supplement arm, which was not surprising considering the late stage in presentation of the disease. One patient died after being admitted to hospital with suspected gastric carcinoma less than 4 months after initiating ART. Patients with gastrointestinal symptoms have been found to have severe intestinal damage and nutrient malabsorption. This may account for the further deterioration in BMI to 14 kg/m^2^. Our results suggest that patients in the NS arm are more likely to be in care by 6 months after ART initiation compared to the Control arm, who were provided standard of care (Hazard ratio 1.26 95% CI 0.65 – 2.42), however this was not statistically significant and our study had insufficient statistical power to demonstrate this difference. Of concern, are the high rates of loss to initiation and LTFU, both common problems in resource limited settings.

The supplement was well tolerated, as evidenced by the absence of gastrointestinal symptoms and adverse side effects in the nutritional supplement arm. This may have provided an incentive for regular clinic attendance. An adequate diet is an important factor influencing adherence to ART [40]. Studies have reported improved ART adherence among food-insecure patients provided with macronutrients [41]. A recent study in Zambia showed that after 6 months patients receiving food assistance had higher ART adherence compared to those not receiving food assistance (n = 147, 98.3% versus 88.8%, respectively; p < 0.01) [42]. The provision of food assistance to HIV-infected adults on ART may have an incentivizing effect which can improve medication adherence, particularly among patients recently initiated on treatment and those with poor nutrition or advanced disease [42]. We did not observe a significant difference in adherence between the two arms. Both groups were provided excellent medical care and counselling which could have led to changes in patient behaviour (and diet) that reduced the difference between the 2 groups at 6 months. This difference may be more evident in resource-limited settings where the level of care is not the same as a clinical trial setting.

### Strengths and limitations

Our finding should be considered in light of the study limitations. First, our results may also be biased by the number of patients who were excluded in the first days and weeks of the study mainly lost to initiation, sicker patients who required hospitalization, disinterest in the intervention or patients that transferred out or withdrew due to work commitments. Both arms presented equally with self-reported weight-loss and advanced HIV disease. Our findings are not only applicable to malnourished HIV-positive patients (less than 30% of our patients had a BMI < 18.5 kg/m^2^) but also HIV-positive patients that present at ART initiation with self-reported weight loss. Second, as Sattler and colleagues (2009) highlighted, it is important to obtain weight and dietary history and quantification of total energy and macronutrient intakes (by food diaries, food frequency, or 24 h dietary recall questionnaires) before prescribing nutritional supplements for patients with HIV [43]. While we do not report on the protein or caloric intake, the serving sizes of starch, meat or vegetables that patients normally consume, the calorie expenditure (kcal) and calculated daily estimated energy expenditure were similar between the two arms, at ART initiation and at 6 months after ART initiation (results not shown) [43]. Third, bioelectrical impedance measurements were made by different examiners at the same clinic which may have resulted in inter-observer variation. We limited this by training experienced nurses and study coordinators to administer questionnaires, measure height, weight, wrist circumference and to record the BIA. Study staff were trained using standardized procedures and measurements were repeated twice and the average used for analysis. Fourth, a limitation of the study was the relatively small sample size. Consistent with other pilot studies, wider investigation with a larger sample size, increased duration of intervention and longer follow-up to determine the impact on patient treatment outcomes such as mortality is recommended [28]. Lastly, we report an improvement in physical activity – this study was performed in patients with inadequate nutritional intake, on-going weight loss and pre-existing malnutrition so the increased intake may lead to a net increase in energy intake on the NS arm with subsequent improvement in physical activity.

Despite these limitations, our work contributes to the discourse on use of macronutrients in HIV-infected populations. Despite the availability, the uptake, quality and effectiveness of nutritional support services and their impact on patient and program outcomes still needs to be determined [44].

## Conclusion

Nutritional supplementation is a feasible method to restore food energy intake in HIV-infected patients with recent weight loss. Benefits of nutritional supplementation included a significant increase in body weight, correcting changes in body composition and improving immune function in HIV-positive patients that present at ART initiation with weight loss. Additional benefits of nutritional supplementation include reversing malnutrition, reducing inflammation or severity of anemia (improvement in red blood cell count and hemoglobin levels) and improving physical activity, thereby improving quality of life and ultimately reducing HIV-associated complications. Larger studies with long-term follow-up are necessary to validate these data.

## Competing interests

The authors have declared that no competing interests exist. Right to Care (RTC) provided some of the funding for the current research and also supports the provision of treatment for the patients in the study.

## Authors’ contributions

IS, MM and DE contributed to study design, implementation of the study and supervised data collection. LM performed data management. DE performed statistical analysis and wrote the first draft of the manuscript. TW, KS, DvA, NB assisted with the design of study, facilitated the implementation of the study, responsible for data collection and verification and also provided patient care to study participants. IS, MM, DE and LM critically reviewed and revised the manuscript. The final version of the paper was reviewed and approved by all authors. All authors read and approved the final manuscript.
